# Crystal structure of racemic [(1*R*,2*S*,3*R*,4*S*,6*S*)-2,6-bis­(furan-2-yl)-4-hy­droxy-4-(thio­phen-2-yl)cyclo­hexane-1,3-di­yl]bis­(thio­phen-2-yl­methanone)

**DOI:** 10.1107/S2056989016009452

**Published:** 2016-06-17

**Authors:** Ísmail Çelik, Cem Cüneyt Ersanlı, Mehmet Akkurt, Hayrettin Gezegen, Rahmi Köseoğlu

**Affiliations:** a Department of Physics, Faculty of Sciences, Cumhuriyet University, 58140 Sivas, Turkey; b Department of Physics, Faculty of Arts and Sciences, Sinop University, 57010 Sinop, Turkey; c Department of Physics, Faculty of Sciences, Erciyes University, 38039 Kayseri, Turkey; d Department of Chemistry, Faculty of Sciences, Cumhuriyet University, 58140 Sivas, Turkey

**Keywords:** crystal structure, domino reactions, penta­substituted cyclo­hexa­nol derivatives

## Abstract

In the title compound, C_28_H_22_O_5_S_3_, the central cyclo­hexane ring adopts a chair conformation. The atoms of the furan ring attached to the 6-position of the central cyclo­hexane ring are disordered over two sets of sites with occupancies of 0.832 (5) and 0.168 (5). The hy­droxy group is disordered over two positions (at the 4- and 6-positions of the cyclo­hexane ring) in the ratio 0.832 (5):0.168 (5). In the crystal, mol­ecules are linked by C—H⋯O hydrogen bonds and C—H⋯π inter­actions, forming layers parallel to (100).

## Abstract 2

The central cyclo­hexane ring has a chair conformation. In the crystal, mol­ecules are linked by C—H⋯O hydrogen bonds and C—H⋯π inter­actions, forming layers parallel to (100).

## Chemical context   

Domino or cascade reactions have many applications in organic chemistry (Tietze *et al.*, 2006 ▸). They are used for the synthesis of complex mol­ecules that have polysubstituted and multiple stereocenters in a single step (Pellissier, 2012 ▸, 2013 ▸). Penta­substituted cyclo­hexa­nol derivatives can be synthesized from aromatic aldehydes and ketones *via* domino reaction (Luo & Shan, 2006 ▸; Gezegen & Ceylan, 2015 ▸). In this paper we report the synthesis of [(1*R*,2*S*,3*R*,4*S*,6*S*)-2,6-bis­(furan-2-yl)-4-hy­droxy-4-(thio­phen-2-yl)cyclo­hexane-1,3-di­yl]bis­(thio­phen-2-yl­methanone) in a high yield starting from 2-acetyl­thio­phene and furfural. The resulting product is a racemate crystallizing in a centrosymmetric space group.
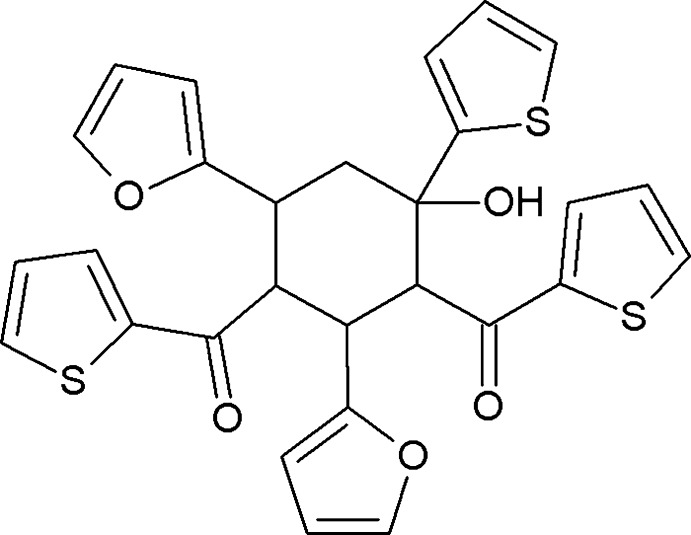


## Structural commentary   

**Figure 1:**
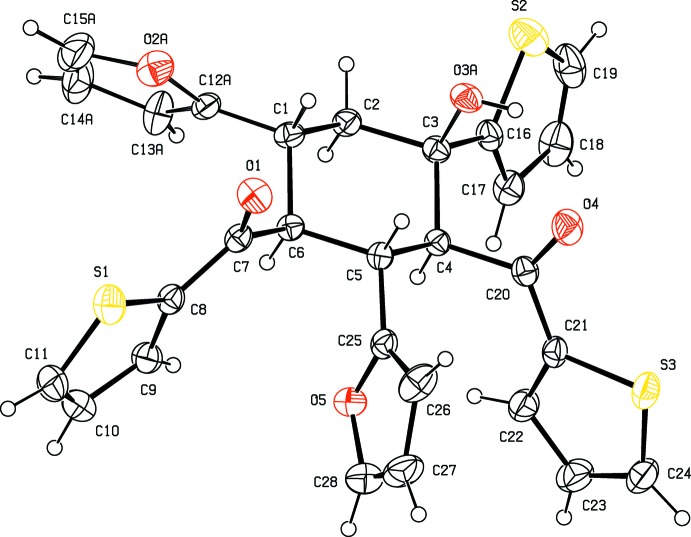
View of the title compound with the atom-numbering scheme. Displacement ellipsoids for non-H atoms are drawn at the 30% probability level. The minor disorder component is not shown, for clarity.

In the title compound, Fig. 1 ▸, the central cyclo­hexane ring adopts a chair conformation [the puckering parameters are *Q*_T_ = 0.586 (3) Å, θ = 0.0 (3)° and φ = 169 (17)°]. The mean plane of this ring makes dihedral angles of 80.42 (14), 59.57 (17), 85.65 (17), 66.82 (19), 84.88 (18) and 83.1 (8)°, respectively, with the five associated five-membered rings (S1/C8–C11, S2/C16–C19, S3/C21–C24, O2*A*/C12*A*–C15*A*, O5/C25–C28 and O2*B*/C12*B*–C15*B*).

## Supra­molecular features   

**Figure 2:**
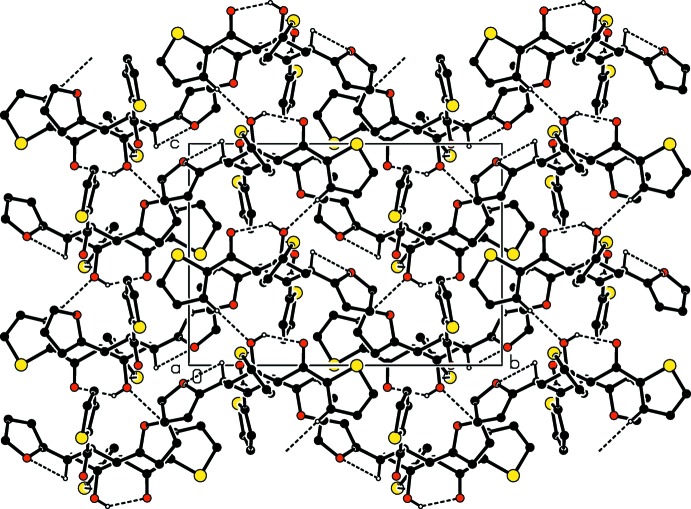
View of the packing of the title compound down the *a* axis. For clarity, the minor disorder component is not shown.

The mol­ecular conformation is stabilized by a weak intra­molecular O—H⋯O inter­action (Table 1 ▸). C—H⋯O hydrogen bonds together with C—H⋯π contacts, Table 1 ▸, form layers of mol­ecules when viewed along both the *a-* and *b-*axis directions, Figs. and 3. Short S3⋯S3^ii^ contacts [symmetry code: (ii) −*x* + 1, −*y*, −*z* + 1] at 3.5210 (12) Å may also contribute to the crystal packing (Figs. 2 ▸ and 3 ▸).

## Semi-empirical quantum mechanical calculations   

**Figure 4:**
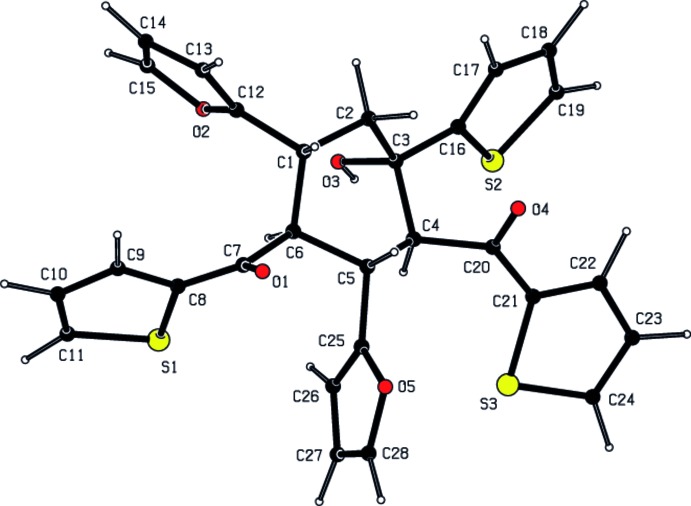
Spatial view of the mol­ecule of the title compound calculated using the *CNDO* method.

According to the results of theoretical calculations carried out using the semi-empirical quantum-mechanical *CNDO/2* (*Complete Neglect of Differential Overlap*) method (Pople & Beveridge, 1970 ▸), the spatial view of the single mol­ecule, with atomic labels, calculated as a closed-shell in a vacuum is shown in Fig. 4 ▸. The net charges on atoms O1, O2, O3, O4, O5, S1, S2 and S3 are −0.330, −0.113, −0.279, −0.341, −0.158, −0.014, −0.055 and −0.021 e^−^, respectively. This is useful as prediction of the electron-rich and electron-poor sites of a molecule provides a rough estimate of chemical and physical properties of the molecule. The dipole moment of the title mol­ecule is 3.626 Debye. The *HOMO* and *LUMO* energy levels are −10.31 and 1.72 eV, respectively. The values of the *HOMO* and *LUMO* energy levels determine the way in which the molecule interacts with other species and help to characterize the chemical reactivity and kinetics of the molecule.

The geometrical parameter values obtained by the X-ray structure determination of the title compound are consistent with those calculated by the CNDO/2 method within the error limits (Table 2 ▸). Small differences between the theoretical and experimental results may result from the calculations assuming the mol­ecule is in a vacuum.

## Synthesis and crystallization   

[(1*R*,2*S*,3*R*,4*S*,6*S*)-2,6-bis­(furan-2-yl)-4-hy­droxy-4-(thio­phen-2-yl)cyclo­hexane-1,3-di­yl]bis­(thio­phen-2-yl­methanone) was synthesized according to a literature method (Gezegen & Ceylan, 2015 ▸) in 87% yield. Colourless prisms were recrystallized from ethanol solution, m.p = 524–526 K.

## Refinement details   

Crystal data, data collection and structure refinement details are summarized in Table 3 ▸. Atoms O2,C12-C15 atoms of the furan ring bound to the C1 atom of the central cyclo­hexane ring are disordered over two sets of sites with an occupancy ratio 0.832 (5):0.168 (5). The O3 hy­droxy group is disordered over two positions (on the C1 and C3 atoms of the cyclo­hexane ring) in the same ratio. The positionally disordered H atoms (H1*A* on C1 and H1*B* on C3) were found from a difference Fourier map and their positions were constrained to the expected geometries [C—H = 0.95±0.02 Å] with a fixed *U* value of 0.05 Å^2^. All other H atoms were placed in calculated positions (C—H = 0.93–0.98, O—H = 0.82 Å) and refined using a riding model with *U*_iso_(H) = 1.2*U*_eq_(carrier).

## Supplementary Material

Crystal structure: contains datablock(s) global, I. DOI: 10.1107/S2056989016009452/sj5500sup1.cif

Structure factors: contains datablock(s) I. DOI: 10.1107/S2056989016009452/sj5500Isup2.hkl

Supporting information file. DOI: 10.1107/S2056989016009452/sj5500Isup3.cml

CCDC reference: 1484675

Additional supporting information: crystallographic information; 3D view; checkCIF report

## Figures and Tables

**Figure 3:**
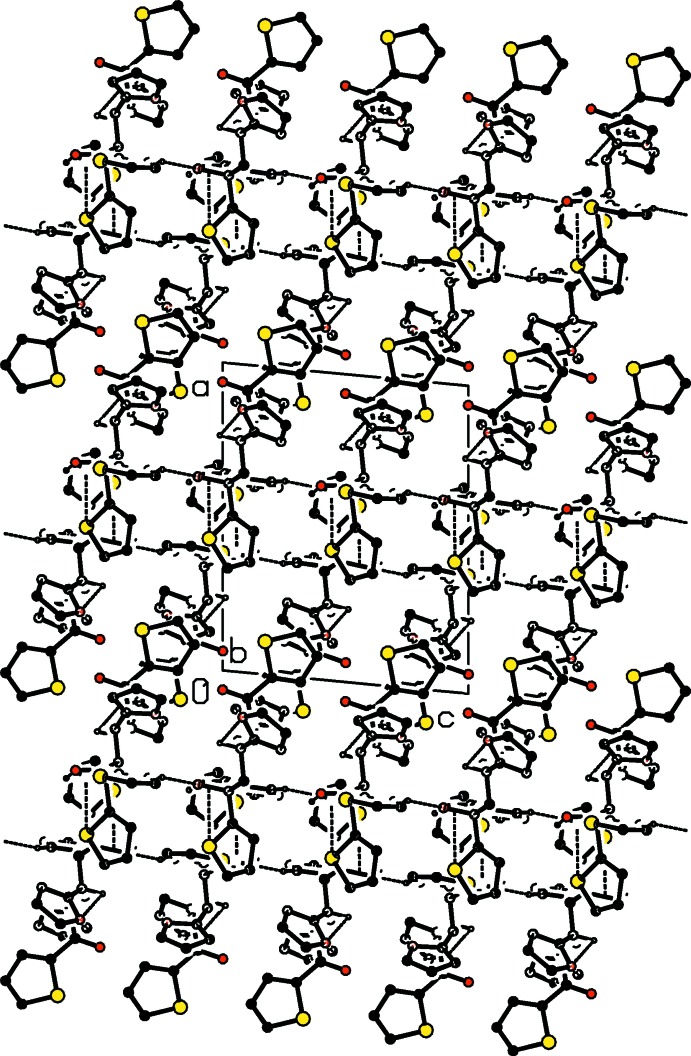
View of the packing of the title compound down the *b*-axis. For clarity, the minor disorder component is not shown.

**Table 1:** Hydrogen-bond geometry (Å, °) *Cg*1 and *Cg*2 are the centroids of the S1/C8–C11 and S2/C16–C19 thio­phene rings, respectively.

*D*—H⋯*A*	*D*—H	H⋯*A*	*D*⋯*A*	*D*—H⋯*A*
O3*A*—H3*A*⋯O4	0.82	2.08	2.673 (4)	129
C22—H22⋯O3*A* ^i^	0.93	2.40	3.321 (4)	170
C24—H24⋯*Cg*2^ii^	0.93	2.95	3.650 (4)	133
C27—H27⋯*Cg*1^iii^	0.93	2.89	3.612 (4)	135
C14*B*—H14*B*⋯*Cg*1^iv^	0.93	2.87	3.772 (19)	163

Symmetry codes: (i) 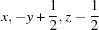; (ii) 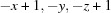; (iii) 
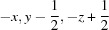
; (iv) 
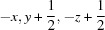
.

**Table 2:** Comparison of geometrical parameters from the X-ray study and semi-empirical quantum-mechanical *CNDO/2* calculations (Å, °)

Bond	X-ray	*CNDO/2*
S1—C8	1.720 (3)	1.771
S1—C11	1.698 (4)	1.767
S2—C16	1.713 (3)	1.778
S2—C19	1.673 (5)	1.763
S3—C21	1.726 (3)	1.769
S3—C24	1.691 (4)	1.765
O1—C7	1.223 (4)	1.213
O2*A*—C12*A*	1.420 (5)	1.360
O2*A*—C15*A*	1.420 (5)	1.358
O3*A*—C3	1.445 (3)	1.420
O4—C20	1.231 (4)	1.214
O5—C28	1.358 (5)	1.356
O5—C25	1.383 (4)	1.360
		
C2—C1—C12*A*	112.8 (8)	112.2
C2—C1—C6	110.2 (2)	113.3
C6—C1—C12*A*	112.0 (3)	111.2
C2—C3—C16	109.8 (2)	113.6
C2—C3—C4	109.4 (2)	110.6
C4—C3—C16	111.4 (2)	110.7
O3*A*—C3—C16	109.6 (2)	106.6
C3—C4—C20	111.8 (2)	111.3
C5—C4—C20	107.2 (2)	109.4
C3—C4—C5	110.5 (2)	112.8
C6—C5—C25	112.4 (2)	111.3
C4—C5—C25	110.5 (2)	111.9
C4—C5—C6	111.2 (2)	112.5
C5—C6—C7	108.6 (2)	113.1
C1—C6—C7	109.9 (2)	109.9
C1—C6—C5	110.6 (2)	107.9
O1—C7—C8	120.4 (3)	121.8
C6—C7—C8	120.2 (3)	118.2
O1—C7—C6	119.4 (3)	119.9
O4—C20—C4	119.4 (3)	119.1
C4—C20—C21	119.7 (3)	119.6
O4—C20—C21	120.8 (3)	121.2

**Table 3:** Experimental details

Crystal data	
Chemical formula	C_28_H_22_O_5_S_3_
*M*_r_	534.64
Crystal system, space group	Monoclinic, *P*2_1_/*c*
Temperature (K)	296
*a*, *b*, *c* (Å)	14.0915 (11), 15.8984 (12), 11.2964 (7)
β (°)	95.421 (2)
*V* (Å^3^)	2519.4 (3)
*Z*	4
Radiation type	Mo *K*α
μ (mm^−1^)	0.33
Crystal size (mm)	0.19 × 0.17 × 0.13

Data collection	
Diffractometer	Bruker APEXII CCD
Absorption correction	Multi-scan (*SADABS*; Sheldrick, 2003 ▸)
*T*_min_, *T*_max_	0.597, 0.746
No. of measured, independent and observed [*I* > 2σ(*I*)] reflections	52585, 6252, 3960
*R*_int_	0.070
(sin θ/λ)_max_ (Å^−1^)	0.668

Refinement	
*R*[*F* ^2^ > 2σ(*F* ^2^)], *wR*(*F* ^2^), *S*	0.066, 0.204, 1.04
No. of reflections	6252
No. of parameters	338
No. of restraints	6
H-atom treatment	H atoms treated by a mixture of independent and constrained refinement
Δρ_max_, Δρ_min_ (e Å^−3^)	0.75, −0.74

Computer programs: *APEX2* and *SAINT* (Bruker, 2007 ▸), *SHELXS97* (Sheldrick, 2008 ▸), *SHELXL2014* (Sheldrick, 2015 ▸), *ORTEP-3 for Windows* (Farrugia, 2012 ▸) and *PLATON* (Spek, 2003 ▸).

## supplementary crystallographic information

### Crystal data

**Table:**

C_28_H_22_O_5_S_3_	*F*(000) = 1112
*M**_r_* = 534.64	*D*_x_ = 1.410 Mg m^−^^3^
Monoclinic, *P*2_1_/*c*	Mo *K*α radiation, λ = 0.71073 Å
Hall symbol: -P 2ybc	Cell parameters from 9982 reflections
*a* = 14.0915 (11) Å	θ = 3.1–28.2°
*b* = 15.8984 (12) Å	µ = 0.33 mm^−^^1^
*c* = 11.2964 (7) Å	*T* = 296 K
β = 95.421 (2)°	Prism, colourless
*V* = 2519.4 (3) Å^3^	0.19 × 0.17 × 0.13 mm
*Z* = 4	

### Data collection

**Table:**

Bruker APEXII CCD diffractometer	3960 reflections with *I* > 2σ(*I*)
φ and ω scans	*R*_int_ = 0.070
Absorption correction: multi-scan (SADABS; Sheldrick, 2003)	θ_max_ = 28.4°, θ_min_ = 3.1°
*T*_min_ = 0.597, *T*_max_ = 0.746	*h* = −18→18
52585 measured reflections	*k* = −21→21
6252 independent reflections	*l* = −15→13

### Refinement

**Table:**

Refinement on *F*^2^	6 restraints
Least-squares matrix: full	Hydrogen site location: mixed
*R*[*F*^2^ > 2σ(*F*^2^)] = 0.066	H atoms treated by a mixture of independent and constrained refinement
*wR*(*F*^2^) = 0.204	*w* = 1/[σ^2^(*F*_o_^2^) + (0.101*P*)^2^ + 2.3401*P*] where *P* = (*F*_o_^2^ + 2*F*_c_^2^)/3
*S* = 1.04	(Δ/σ)_max_ < 0.001
6252 reflections	Δρ_max_ = 0.75 e Å^−^^3^
338 parameters	Δρ_min_ = −0.74 e Å^−^^3^

### Special details

**Table:**

Geometry. Bond distances, angles etc. have been calculated using the rounded fractional coordinates. All su's are estimated from the variances of the (full) variance-covariance matrix. The cell esds are taken into account in the estimation of distances, angles and torsion angles
Refinement. Refinement on F^2^ for ALL reflections except those flagged by the user for potential systematic errors. Weighted R-factors wR and all goodnesses of fit S are based on F^2^, conventional R-factors R are based on F, with F set to zero for negative F^2^. The observed criterion of F^2^ > 2sigma(F^2^) is used only for calculating -R-factor-obs etc. and is not relevant to the choice of reflections for refinement. R-factors based on F^2^ are statistically about twice as large as those based on F, and R-factors based on ALL data will be even larger.

### Fractional atomic coordinates and isotropic or equivalent isotropic displacement parameters (Å^2^)

**Table:**

	*x*	*y*	*z*	*U*_iso_*/*U*_eq_	Occ. (<1)
S1	−0.10680 (6)	0.34243 (6)	0.32727 (9)	0.0528 (3)	
S2	0.57614 (7)	0.33394 (7)	0.55457 (10)	0.0661 (4)	
S3	0.38176 (6)	−0.03610 (5)	0.49813 (8)	0.0497 (3)	
O1	0.06307 (16)	0.33764 (15)	0.50045 (19)	0.0468 (7)	
O2A	0.1462 (2)	0.51774 (19)	0.4184 (3)	0.0541 (10)	0.832 (5)
O2B	0.2243 (12)	0.4730 (9)	0.2304 (15)	0.0541 (10)	0.168 (5)
O3A	0.3783 (2)	0.30198 (19)	0.6008 (2)	0.0419 (8)	0.832 (5)
O3B	0.2474 (13)	0.4000 (12)	0.5522 (11)	0.050 (5)	0.168 (5)
O4	0.37016 (18)	0.13400 (15)	0.59929 (19)	0.0516 (8)	
O5	0.16291 (18)	0.14210 (14)	0.2732 (2)	0.0522 (8)	
C1	0.2479 (2)	0.38831 (18)	0.4265 (3)	0.0354 (9)	
C2	0.3529 (2)	0.37285 (18)	0.4141 (3)	0.0360 (9)	
C3	0.3902 (2)	0.29228 (18)	0.4759 (2)	0.0339 (8)	
C4	0.33066 (19)	0.21646 (17)	0.4248 (2)	0.0302 (8)	
C5	0.22355 (19)	0.23054 (17)	0.4381 (2)	0.0317 (8)	
C6	0.18765 (19)	0.31239 (16)	0.3769 (2)	0.0304 (8)	
C7	0.0835 (2)	0.32507 (17)	0.3991 (3)	0.0335 (8)	
C8	0.0088 (2)	0.32100 (18)	0.3002 (3)	0.0354 (9)	
C9	0.0139 (2)	0.3006 (2)	0.1833 (3)	0.0433 (10)	
C10	−0.0750 (3)	0.3032 (2)	0.1164 (3)	0.0578 (12)	
C11	−0.1458 (3)	0.3250 (2)	0.1826 (4)	0.0592 (14)	
C12A	0.2159 (3)	0.46624 (16)	0.3713 (3)	0.0372 (10)	0.832 (5)
C12B	0.2063 (16)	0.4706 (10)	0.3519 (15)	0.0372 (10)	0.168 (5)
C13A	0.2394 (3)	0.5031 (3)	0.2634 (3)	0.0602 (17)	0.832 (5)
C13B	0.1550 (17)	0.5444 (12)	0.3766 (17)	0.0602 (17)	0.168 (5)
C14A	0.1842 (3)	0.5774 (2)	0.2437 (4)	0.089 (3)	0.832 (5)
C14B	0.1413 (19)	0.5924 (11)	0.270 (2)	0.089 (3)	0.168 (5)
C15A	0.1266 (3)	0.58644 (17)	0.3395 (4)	0.095 (3)	0.832 (5)
C15B	0.1841 (17)	0.5483 (12)	0.1800 (16)	0.095 (3)	0.168 (5)
C16	0.4946 (2)	0.28136 (19)	0.4597 (3)	0.0368 (9)	
C17	0.5386 (2)	0.2391 (2)	0.3737 (3)	0.0446 (10)	
C18	0.6395 (3)	0.2524 (3)	0.3902 (4)	0.0607 (14)	
C19	0.6665 (3)	0.3005 (3)	0.4807 (4)	0.0648 (15)	
C20	0.3597 (2)	0.13466 (19)	0.4899 (3)	0.0349 (9)	
C21	0.3687 (2)	0.05818 (18)	0.4227 (3)	0.0364 (9)	
C22	0.3728 (3)	0.0465 (2)	0.3024 (3)	0.0470 (11)	
C23	0.3884 (3)	−0.0380 (2)	0.2741 (4)	0.0588 (14)	
C24	0.3939 (3)	−0.0894 (2)	0.3709 (4)	0.0564 (13)	
C25	0.1672 (2)	0.15642 (18)	0.3943 (3)	0.0364 (9)	
C26	0.1219 (3)	0.0946 (2)	0.4462 (4)	0.0583 (14)	
C27	0.0863 (3)	0.0390 (2)	0.3511 (4)	0.0651 (14)	
C28	0.1138 (3)	0.0694 (3)	0.2513 (4)	0.0681 (16)	
H1A	0.237 (5)	0.395 (4)	0.511 (2)	0.0500*	0.832 (5)
H1B	0.38 (2)	0.310 (18)	0.557 (10)	0.0500*	0.168 (5)
H2A	0.38950	0.42030	0.44750	0.0430*	
H2B	0.36240	0.36950	0.33030	0.0430*	
H3A	0.39870	0.26000	0.63700	0.0630*	0.832 (5)
H3B	0.26770	0.35740	0.58690	0.0750*	0.168 (5)
H4	0.33970	0.21010	0.34030	0.0360*	
H5	0.21640	0.23630	0.52310	0.0380*	
H6	0.19240	0.30770	0.29120	0.0370*	
H9	0.07030	0.28640	0.15160	0.0520*	
H10	−0.08410	0.29140	0.03550	0.0690*	
H11	−0.20920	0.32990	0.15220	0.0710*	
H13A	0.28330	0.48230	0.21420	0.0720*	0.832 (5)
H13B	0.13400	0.55880	0.44960	0.0720*	0.168 (5)
H14A	0.18560	0.61380	0.17940	0.1060*	0.832 (5)
H14B	0.10970	0.64380	0.26150	0.1060*	0.168 (5)
H15A	0.08360	0.62980	0.34890	0.1140*	0.832 (5)
H15B	0.18560	0.56560	0.10150	0.1140*	0.168 (5)
H17	0.50730	0.20670	0.31360	0.0540*	
H18	0.68180	0.22900	0.34100	0.0730*	
H19	0.72980	0.31490	0.50210	0.0780*	
H22	0.36600	0.08960	0.24650	0.0560*	
H23	0.39440	−0.05700	0.19730	0.0710*	
H24	0.40330	−0.14720	0.36740	0.0670*	
H26	0.11480	0.08860	0.52680	0.0700*	
H27	0.05050	−0.00950	0.35890	0.0780*	
H28	0.10130	0.04470	0.17690	0.0820*	

### Atomic displacement parameters (Å^2^)

**Table:**

	*U*^11^	*U*^22^	*U*^33^	*U*^12^	*U*^13^	*U*^23^
S1	0.0311 (4)	0.0639 (6)	0.0647 (6)	0.0092 (4)	0.0107 (4)	0.0048 (4)
S2	0.0504 (6)	0.0790 (7)	0.0673 (7)	−0.0109 (5)	−0.0031 (5)	0.0003 (5)
S3	0.0463 (5)	0.0395 (4)	0.0629 (6)	0.0047 (3)	0.0033 (4)	0.0151 (4)
O1	0.0406 (12)	0.0624 (14)	0.0392 (12)	0.0037 (11)	0.0140 (10)	−0.0035 (10)
O2A	0.0456 (16)	0.0549 (18)	0.064 (2)	0.0046 (14)	0.0166 (14)	−0.0075 (15)
O2B	0.0456 (16)	0.0549 (18)	0.064 (2)	0.0046 (14)	0.0166 (14)	−0.0075 (15)
O3A	0.0450 (15)	0.0512 (16)	0.0295 (13)	0.0019 (12)	0.0033 (13)	−0.0089 (13)
O3B	0.055 (9)	0.060 (9)	0.035 (7)	0.003 (7)	0.007 (7)	−0.011 (8)
O4	0.0655 (16)	0.0536 (14)	0.0353 (12)	0.0054 (12)	0.0029 (11)	0.0087 (10)
O5	0.0627 (16)	0.0415 (12)	0.0497 (13)	−0.0028 (11)	−0.0083 (11)	−0.0046 (10)
C1	0.0337 (15)	0.0335 (14)	0.0395 (16)	−0.0014 (12)	0.0065 (13)	−0.0067 (12)
C2	0.0298 (14)	0.0328 (14)	0.0454 (17)	−0.0019 (11)	0.0034 (12)	−0.0019 (12)
C3	0.0315 (14)	0.0365 (15)	0.0335 (15)	−0.0007 (11)	0.0028 (12)	−0.0015 (12)
C4	0.0284 (14)	0.0320 (14)	0.0303 (14)	0.0006 (11)	0.0032 (11)	−0.0003 (11)
C5	0.0306 (14)	0.0325 (14)	0.0322 (14)	0.0012 (11)	0.0044 (11)	0.0010 (11)
C6	0.0282 (14)	0.0313 (13)	0.0325 (14)	0.0007 (11)	0.0067 (11)	−0.0026 (11)
C7	0.0309 (14)	0.0301 (14)	0.0403 (16)	0.0012 (11)	0.0084 (12)	0.0005 (11)
C8	0.0278 (14)	0.0330 (14)	0.0458 (17)	0.0023 (11)	0.0061 (12)	0.0022 (12)
C9	0.0369 (16)	0.0456 (18)	0.0470 (18)	0.0050 (14)	0.0026 (14)	−0.0055 (14)
C10	0.056 (2)	0.059 (2)	0.055 (2)	0.0027 (18)	−0.0126 (18)	−0.0101 (17)
C11	0.0371 (19)	0.059 (2)	0.078 (3)	0.0043 (16)	−0.0125 (18)	0.0010 (19)
C12A	0.0323 (18)	0.0281 (14)	0.051 (2)	0.0009 (12)	0.0032 (16)	−0.0054 (14)
C12B	0.0323 (18)	0.0281 (14)	0.051 (2)	0.0009 (12)	0.0032 (16)	−0.0054 (14)
C13A	0.060 (3)	0.052 (3)	0.070 (3)	0.014 (2)	0.014 (2)	0.022 (2)
C13B	0.060 (3)	0.052 (3)	0.070 (3)	0.014 (2)	0.014 (2)	0.022 (2)
C14A	0.069 (4)	0.054 (3)	0.137 (6)	−0.006 (3)	−0.020 (4)	0.041 (3)
C14B	0.069 (4)	0.054 (3)	0.137 (6)	−0.006 (3)	−0.020 (4)	0.041 (3)
C15A	0.063 (4)	0.047 (3)	0.167 (7)	0.011 (3)	−0.032 (4)	−0.013 (4)
C15B	0.063 (4)	0.047 (3)	0.167 (7)	0.011 (3)	−0.032 (4)	−0.013 (4)
C16	0.0291 (14)	0.0404 (16)	0.0402 (16)	−0.0010 (12)	0.0001 (12)	0.0064 (12)
C17	0.0301 (16)	0.0478 (18)	0.057 (2)	0.0030 (13)	0.0097 (14)	0.0030 (15)
C18	0.0379 (19)	0.067 (2)	0.080 (3)	0.0084 (17)	0.0205 (18)	0.017 (2)
C19	0.0372 (19)	0.078 (3)	0.077 (3)	−0.0060 (18)	−0.0054 (18)	0.025 (2)
C20	0.0290 (14)	0.0395 (15)	0.0360 (16)	−0.0002 (12)	0.0028 (12)	0.0034 (12)
C21	0.0291 (14)	0.0351 (15)	0.0446 (17)	0.0043 (12)	0.0015 (12)	0.0058 (12)
C22	0.057 (2)	0.0389 (17)	0.0451 (18)	0.0042 (15)	0.0043 (15)	0.0027 (14)
C23	0.069 (3)	0.046 (2)	0.062 (2)	0.0062 (18)	0.0095 (19)	−0.0109 (17)
C24	0.049 (2)	0.0344 (17)	0.085 (3)	0.0054 (15)	0.0028 (18)	0.0012 (17)
C25	0.0279 (14)	0.0341 (14)	0.0469 (17)	0.0036 (12)	0.0027 (12)	0.0012 (12)
C26	0.053 (2)	0.0427 (19)	0.083 (3)	−0.0021 (16)	0.0257 (19)	0.0041 (18)
C27	0.057 (2)	0.0389 (19)	0.099 (3)	−0.0146 (17)	0.005 (2)	−0.007 (2)
C28	0.070 (3)	0.049 (2)	0.081 (3)	−0.0066 (19)	−0.016 (2)	−0.010 (2)

### Geometric parameters (Å, º)

**Table:**

S1—C8	1.720 (3)	C14A—C15A	1.420 (6)
S1—C11	1.697 (5)	C14B—C15B	1.42 (3)
S2—C16	1.713 (3)	C16—C17	1.376 (4)
S2—C19	1.674 (4)	C17—C18	1.432 (5)
S3—C21	1.725 (3)	C18—C19	1.304 (6)
S3—C24	1.691 (4)	C20—C21	1.445 (4)
O1—C7	1.223 (4)	C21—C22	1.378 (5)
O2A—C12A	1.420 (5)	C22—C23	1.403 (5)
O2A—C15A	1.420 (5)	C23—C24	1.361 (6)
O2B—C12B	1.42 (2)	C25—C26	1.337 (5)
O2B—C15B	1.42 (2)	C26—C27	1.444 (6)
O3A—C3	1.445 (3)	C27—C28	1.318 (6)
O3B—C1	1.433 (13)	C1—H1A	0.99 (3)
O4—C20	1.231 (4)	C2—H2A	0.9700
O5—C25	1.383 (4)	C2—H2B	0.9700
O5—C28	1.358 (5)	C3—H1B	0.98 (16)
O3A—H3A	0.8200	C4—H4	0.9800
O3B—H3B	0.8200	C5—H5	0.9800
C1—C2	1.519 (4)	C6—H6	0.9800
C1—C12B	1.635 (17)	C9—H9	0.9300
C1—C12A	1.440 (4)	C10—H10	0.9300
C1—C6	1.550 (4)	C11—H11	0.9300
C2—C3	1.528 (4)	C13A—H13A	0.9300
C3—C16	1.510 (4)	C13B—H13B	0.9300
C3—C4	1.549 (4)	C14A—H14A	0.9300
C4—C5	1.547 (4)	C14B—H14B	0.9300
C4—C20	1.531 (4)	C15A—H15A	0.9300
C5—C6	1.537 (4)	C15B—H15B	0.9300
C5—C25	1.480 (4)	C17—H17	0.9300
C6—C7	1.526 (4)	C18—H18	0.9300
C7—C8	1.462 (4)	C19—H19	0.9300
C8—C9	1.368 (5)	C22—H22	0.9300
C9—C10	1.401 (5)	C23—H23	0.9300
C10—C11	1.348 (6)	C24—H24	0.9300
C12A—C13A	1.420 (5)	C26—H26	0.9300
C12B—C13B	1.42 (3)	C27—H27	0.9300
C13A—C14A	1.420 (6)	C28—H28	0.9300
C13B—C14B	1.42 (3)		
			
C8—S1—C11	91.29 (19)	C21—C22—C23	112.1 (3)
C16—S2—C19	91.72 (19)	C22—C23—C24	113.0 (4)
C21—S3—C24	91.73 (17)	S3—C24—C23	112.3 (3)
C12A—O2A—C15A	108.0 (3)	O5—C25—C26	109.6 (3)
C12B—O2B—C15B	108.0 (15)	C5—C25—C26	134.6 (3)
C25—O5—C28	107.1 (3)	O5—C25—C5	115.7 (2)
C3—O3A—H3A	109.00	C25—C26—C27	105.7 (4)
C1—O3B—H3B	109.00	C26—C27—C28	107.6 (3)
O3B—C1—C12B	111.8 (10)	O5—C28—C27	110.0 (4)
O3B—C1—C2	102.0 (8)	C2—C1—H1A	110 (4)
O3B—C1—C6	113.9 (8)	C6—C1—H1A	108 (4)
C2—C1—C12B	112.8 (8)	C12A—C1—H1A	105 (4)
C6—C1—C12A	112.0 (3)	C1—C2—H2A	109.00
C6—C1—C12B	106.2 (7)	C1—C2—H2B	109.00
C2—C1—C12A	111.5 (3)	C3—C2—H2A	109.00
C2—C1—C6	110.2 (2)	C3—C2—H2B	109.00
C1—C2—C3	112.9 (2)	H2A—C2—H2B	108.00
O3A—C3—C2	106.9 (2)	C2—C3—H1B	96 (14)
C2—C3—C4	109.4 (2)	C4—C3—H1B	117 (15)
O3A—C3—C4	109.8 (2)	C16—C3—H1B	112 (17)
O3A—C3—C16	109.6 (2)	C3—C4—H4	109.00
C4—C3—C16	111.4 (2)	C5—C4—H4	109.00
C2—C3—C16	109.8 (2)	C20—C4—H4	109.00
C3—C4—C20	111.8 (2)	C4—C5—H5	107.00
C3—C4—C5	110.5 (2)	C6—C5—H5	108.00
C5—C4—C20	107.2 (2)	C25—C5—H5	107.00
C4—C5—C25	110.5 (2)	C1—C6—H6	109.00
C4—C5—C6	111.2 (2)	C5—C6—H6	109.00
C6—C5—C25	112.4 (2)	C7—C6—H6	109.00
C5—C6—C7	108.6 (2)	C8—C9—H9	124.00
C1—C6—C7	109.9 (2)	C10—C9—H9	124.00
C1—C6—C5	110.6 (2)	C9—C10—H10	124.00
O1—C7—C6	119.4 (3)	C11—C10—H10	124.00
O1—C7—C8	120.4 (3)	S1—C11—H11	124.00
C6—C7—C8	120.2 (3)	C10—C11—H11	124.00
S1—C8—C7	118.8 (2)	C12A—C13A—H13A	126.00
C7—C8—C9	130.4 (3)	C14A—C13A—H13A	126.00
S1—C8—C9	110.8 (2)	C12B—C13B—H13B	126.00
C8—C9—C10	112.8 (3)	C14B—C13B—H13B	126.00
C9—C10—C11	112.3 (3)	C15A—C14A—H14A	126.00
S1—C11—C10	112.8 (3)	C13A—C14A—H14A	126.00
O2A—C12A—C13A	108.0 (3)	C15B—C14B—H14B	126.00
O2A—C12A—C1	122.2 (3)	C13B—C14B—H14B	126.00
C1—C12A—C13A	129.7 (3)	O2A—C15A—H15A	126.00
O2B—C12B—C1	115.6 (13)	C14A—C15A—H15A	126.00
O2B—C12B—C13B	108.0 (15)	C14B—C15B—H15B	126.00
C1—C12B—C13B	136.4 (15)	O2B—C15B—H15B	126.00
C12A—C13A—C14A	108.0 (3)	C18—C17—H17	125.00
C12B—C13B—C14B	107.9 (17)	C16—C17—H17	125.00
C13A—C14A—C15A	108.0 (3)	C17—C18—H18	123.00
C13B—C14B—C15B	108.0 (17)	C19—C18—H18	123.00
O2A—C15A—C14A	108.0 (3)	S2—C19—H19	123.00
O2B—C15B—C14B	108.1 (16)	C18—C19—H19	123.00
S2—C16—C17	111.1 (2)	C23—C22—H22	124.00
C3—C16—C17	130.6 (3)	C21—C22—H22	124.00
S2—C16—C3	118.2 (2)	C22—C23—H23	124.00
C16—C17—C18	110.1 (3)	C24—C23—H23	123.00
C17—C18—C19	113.7 (4)	S3—C24—H24	124.00
S2—C19—C18	113.4 (3)	C23—C24—H24	124.00
O4—C20—C4	119.4 (3)	C27—C26—H26	127.00
O4—C20—C21	120.8 (3)	C25—C26—H26	127.00
C4—C20—C21	119.7 (3)	C26—C27—H27	126.00
S3—C21—C20	118.8 (3)	C28—C27—H27	126.00
S3—C21—C22	110.9 (2)	C27—C28—H28	125.00
C20—C21—C22	130.2 (3)	O5—C28—H28	125.00
			
C11—S1—C8—C7	−179.5 (2)	C3—C4—C20—O4	46.3 (3)
C11—S1—C8—C9	−0.6 (3)	C3—C4—C5—C6	57.1 (2)
C8—S1—C11—C10	0.5 (3)	C5—C4—C20—O4	−75.0 (3)
C19—S2—C16—C3	−175.7 (3)	C20—C4—C5—C25	−55.4 (3)
C19—S2—C16—C17	0.2 (3)	C4—C5—C6—C7	−176.9 (2)
C16—S2—C19—C18	−0.3 (4)	C6—C5—C25—O5	57.1 (3)
C24—S3—C21—C22	0.7 (3)	C6—C5—C25—C26	−127.6 (4)
C24—S3—C21—C20	−176.8 (3)	C25—C5—C6—C7	58.7 (3)
C21—S3—C24—C23	0.0 (3)	C25—C5—C6—C1	179.3 (2)
C15A—O2A—C12A—C1	−176.4 (3)	C4—C5—C25—O5	−67.7 (3)
C12A—O2A—C15A—C14A	0.0 (4)	C4—C5—C6—C1	−56.2 (3)
C15A—O2A—C12A—C13A	0.0 (4)	C4—C5—C25—C26	107.5 (4)
C28—O5—C25—C5	176.3 (3)	C1—C6—C7—O1	−55.6 (3)
C28—O5—C25—C26	−0.2 (4)	C5—C6—C7—O1	65.5 (3)
C25—O5—C28—C27	1.1 (4)	C5—C6—C7—C8	−113.6 (3)
C6—C1—C12A—O2A	90.7 (4)	C1—C6—C7—C8	125.3 (3)
C2—C1—C12A—C13A	39.2 (5)	C6—C7—C8—C9	5.6 (5)
C2—C1—C6—C5	55.4 (3)	O1—C7—C8—C9	−173.5 (3)
C6—C1—C12A—C13A	−84.9 (5)	O1—C7—C8—S1	5.1 (4)
C12A—C1—C6—C7	−59.9 (3)	C6—C7—C8—S1	−175.8 (2)
C12A—C1—C6—C5	−179.8 (3)	S1—C8—C9—C10	0.6 (4)
C12A—C1—C2—C3	177.8 (3)	C7—C8—C9—C10	179.3 (3)
C2—C1—C6—C7	175.3 (2)	C8—C9—C10—C11	−0.2 (4)
C2—C1—C12A—O2A	−145.2 (3)	C9—C10—C11—S1	−0.3 (4)
C6—C1—C2—C3	−57.1 (3)	C1—C12A—C13A—C14A	176.0 (4)
C1—C2—C3—C16	−180.0 (3)	O2A—C12A—C13A—C14A	0.0 (4)
C1—C2—C3—O3A	−61.2 (3)	C12A—C13A—C14A—C15A	0.0 (5)
C1—C2—C3—C4	57.5 (3)	C13A—C14A—C15A—O2A	0.0 (4)
C2—C3—C4—C5	−56.4 (3)	S2—C16—C17—C18	−0.1 (4)
C2—C3—C16—C17	−92.0 (4)	C3—C16—C17—C18	175.2 (3)
O3A—C3—C4—C20	−58.7 (3)	C16—C17—C18—C19	−0.1 (5)
C2—C3—C16—S2	83.0 (3)	C17—C18—C19—S2	0.2 (5)
O3A—C3—C4—C5	60.6 (3)	O4—C20—C21—C22	−170.1 (4)
C16—C3—C4—C20	62.9 (3)	O4—C20—C21—S3	6.8 (4)
C2—C3—C4—C20	−175.6 (2)	C4—C20—C21—C22	13.8 (5)
O3A—C3—C16—C17	150.9 (3)	C4—C20—C21—S3	−169.3 (2)
O3A—C3—C16—S2	−34.1 (3)	S3—C21—C22—C23	−1.3 (4)
C4—C3—C16—C17	29.3 (4)	C20—C21—C22—C23	175.8 (3)
C4—C3—C16—S2	−155.75 (19)	C21—C22—C23—C24	1.3 (5)
C16—C3—C4—C5	−177.9 (2)	C22—C23—C24—S3	−0.8 (5)
C3—C4—C5—C25	−177.4 (2)	O5—C25—C26—C27	−0.7 (4)
C20—C4—C5—C6	179.2 (2)	C5—C25—C26—C27	−176.2 (3)
C5—C4—C20—C21	101.2 (3)	C25—C26—C27—C28	1.4 (5)
C3—C4—C20—C21	−137.6 (3)	C26—C27—C28—O5	−1.5 (5)

### Hydrogen-bond geometry (Å, º)

Cg1 and Cg2 are the centroids of the S1/C8–C11 and S2/C16–C19 thiophene rings, respectively.

**Table:**

*D*—H···*A*	*D*—H	H···*A*	*D*···*A*	*D*—H···*A*
O3*A*—H3*A*···O4	0.82	2.08	2.673 (4)	129
C5—H5···O3*A*	0.98	2.59	2.944 (4)	102
C22—H22···O3*A*^i^	0.93	2.40	3.321 (4)	170
C24—H24···*Cg*2^ii^	0.93	2.95	3.650 (4)	133
C27—H27···*Cg*1^iii^	0.93	2.89	3.612 (4)	135
C14*B*—H14*B*···*Cg*1^iv^	0.93	2.87	3.772 (19)	163

Symmetry codes: (i) *x*, −*y*+1/2, *z*−1/2; (ii) −*x*+1, −*y*, −*z*+1; (iii) −*x*, *y*−1/2, −*z*+1/2; (iv) −*x*, *y*+1/2, −*z*+1/2.
