# Crystal structure of racemic [(1*R*,2*S*,3*R*,4*S*,6*S*)-2,6-bis­(furan-2-yl)-4-hy­droxy-4-(thio­phen-2-yl)cyclo­hexane-1,3-di­yl]bis­(thio­phen-2-yl­methanone). Corrigendum

**DOI:** 10.1107/S2056989016011452

**Published:** 2016-07-29

**Authors:** Ísmail Çelik, Cem Cüneyt Ersanlı, Mehmet Akkurt, Hayreddin Gezegen, Rahmi Köseoğlu

**Affiliations:** a Department of Physics, Faculty of Sciences, Cumhuriyet University, 58140 Sivas, Turkey; b Department of Physics, Faculty of Arts and Sciences, Sinop University, 57010 Sinop, Turkey; c Department of Physics, Faculty of Sciences, Erciyes University, 38039 Kayseri, Turkey; d Department of Nutrition and Dietetics, Faculty of Health Sciences, Cumhuriyet University, 58140 Sivas, Turkey

## Abstract

In the paper by Çelik *et al.* [*Acta Cryst.* (2016), E**72**, 976–979], the name and affiliation of one of the authors is incorrect.

## Abstract 2

Erratum to *Acta Cryst.* (2016), E**72**, 976–979.

In the paper by Çelik *et al.* (2016 ▸), the name and affiliation of the fourth author, Hayreddin Gezegen, was given incorrectly. The correct affiliation should be ‘Department of Nutrition and Dietetics, Faculty of Health Sciences, Cumhuriyet University’, as is given above.
